# Supplementary material for: Predation and fragmentation portrayed in the statistical structure of prey time series
Source: BMC Ecol. 2009 May 6;9:10. doi: 10.1186/1472-6785-9-10 (PMC2689204; doi:10.1186/1472-6785-9-10)
Supplement: Additional file 2 — Voles and related classes ODDox Documentation. ODDox documentation of the agent-based model (ALMaSS) applied by Hendrichsen et al. The documentation is started by activating main.html. [file 1472-6785-9-10-S2.zip › Vole_ODDox/globals_func.html]

ALMaSS ODDox: Class Members

- Main Page
- Related Pages
- Classes
- Files

- File List
- File Members

- All
- Functions
- Variables
- Typedefs
- Enumerations
- Enumerator
- Defines

- c
- l

### - c -

- cfg\_CatastropheEventStartYear()
  : PopulationManager.cpp- cfg\_CIPEGridOutput\_day()
    : PopulationManager.cpp- cfg\_CIPEGridOutput\_day\_b()
      : PopulationManager.cpp- cfg\_CIPEGridOutput\_filename()
        : PopulationManager.cpp- cfg\_CIPEGridOutput\_filenameB()
          : PopulationManager.cpp- cfg\_CIPEGridOutput\_Interval()
            : PopulationManager.cpp- cfg\_CIPEGridOutput\_used()
              : PopulationManager.cpp- cfg\_DayInMonth()
                : PopulationManager.cpp- cfg\_extradispmort()
                  : Vole\_all.cpp- cfg\_f1sterilitychance()
                    : VolePopulationManager.cpp- cfg\_fixed\_random\_sequence()
                      : PopulationManager.cpp- cfg\_geneticproductfertilityeffect()
                        : VolePopulationManager.cpp- cfg\_geneticsterilitychance()
                          : VolePopulationManager.cpp- cfg\_GrassStartGrowth()
                            : VolePopulationManager.cpp- cfg\_MoveToLessFavourable()
                              : VolePopulationManager.cpp- cfg\_organic\_extensive()
                                : farm.cpp- cfg\_owl\_breed\_day()
                                  : Predators.cpp- cfg\_owl\_breed\_threshold()
                                    : Predators.cpp- cfg\_owl\_death\_threshold()
                                      : Predators.cpp- cfg\_owl\_StartingNo()
                                        : Predators.cpp- cfg\_PesticideAccumulationThreshold()
                                          : Vole\_all.cpp- cfg\_pm\_eventfrequency()
                                            : PopulationManager.cpp- cfg\_pm\_eventsize()
                                              : PopulationManager.cpp- cfg\_ReallyBigOutput\_filename()
                                                : PopulationManager.cpp- cfg\_ReallyBigOutput\_used()
                                                  : PopulationManager.cpp- cfg\_RipleysOutput\_day()
                                                    : PopulationManager.cpp- cfg\_RipleysOutput\_filename()
                                                      : PopulationManager.cpp- cfg\_RipleysOutput\_interval()
                                                        : PopulationManager.cpp- cfg\_RipleysOutput\_used()
                                                          : PopulationManager.cpp- cfg\_RipleysOutputFirstYear()
                                                            : PopulationManager.cpp- cfg\_RipleysOutputMonthly\_used()
                                                              : PopulationManager.cpp- cfg\_setaside\_glyphosate()
                                                                : setaside.cpp- cfg\_UseEasyPop()
                                                                  : PopulationManager.cpp- cfg\_vole\_max\_age()
                                                                    : Vole\_all.cpp- cfg\_vole\_starting\_number()
                                                                      : VolePopulationManager.cpp- cfg\_VoleCatastrophe\_interval()
                                                                        : PopulationManager.cpp- cfg\_VoleCatastrophe\_mortality()
                                                                          : PopulationManager.cpp- cfg\_VoleCatastrophe\_on()
                                                                            : PopulationManager.cpp- cfg\_weasel\_breed\_day()
                                                                              : Predators.cpp- cfg\_weasel\_breed\_threshold()
                                                                                : Predators.cpp- cfg\_weasel\_death\_threshold()
                                                                                  : Predators.cpp- cfg\_weasel\_DispersalMax()
                                                                                    : Predators.cpp- cfg\_weasel\_home\_range()
                                                                                      : Predators.cpp- cfg\_weasel\_kill\_efficiency()
                                                                                        : Predators.cpp- cfg\_weasel\_NoFailuresBeforeDispersal()
                                                                                          : Predators.cpp- cfg\_weasel\_search\_area()
                                                                                            : Predators.cpp- cfg\_weasel\_StartingNo()
                                                                                              : Predators.cpp

### - l -

- l\_farm\_cattle\_veg\_reduce()
  : farmfuncs.cpp- l\_farm\_cattle\_veg\_reduce2()
    : farmfuncs.cpp- l\_farm\_event\_do\_it\_prob()
      : farmfuncs.cpp- l\_farm\_herbicide\_kills()
        : farmfuncs.cpp- l\_farm\_insecticide\_kills()
          : farmfuncs.cpp- l\_farm\_pig\_veg\_reduce()
            : farmfuncs.cpp- l\_map\_rotation\_files\_prefix()
              : croprotation.cpp

---

Generated on Thu Jan 22 14:13:48 2009 for ALMaSS ODDox by 
 1.5.6
